# Supplementary material for: RIPK1 Regulates Microglial Activation in Lipopolysaccharide-Induced Neuroinflammation and MPTP-Induced Parkinson’s Disease Mouse Models
Source: Cells. 2023 Jan 26;12(3):417. doi: 10.3390/cells12030417 (PMC9913664; doi:10.3390/cells12030417)
Supplement: Supplementary file 1 [file cells-12-00417-s001.zip › Supplementary Figure S1.pdf]

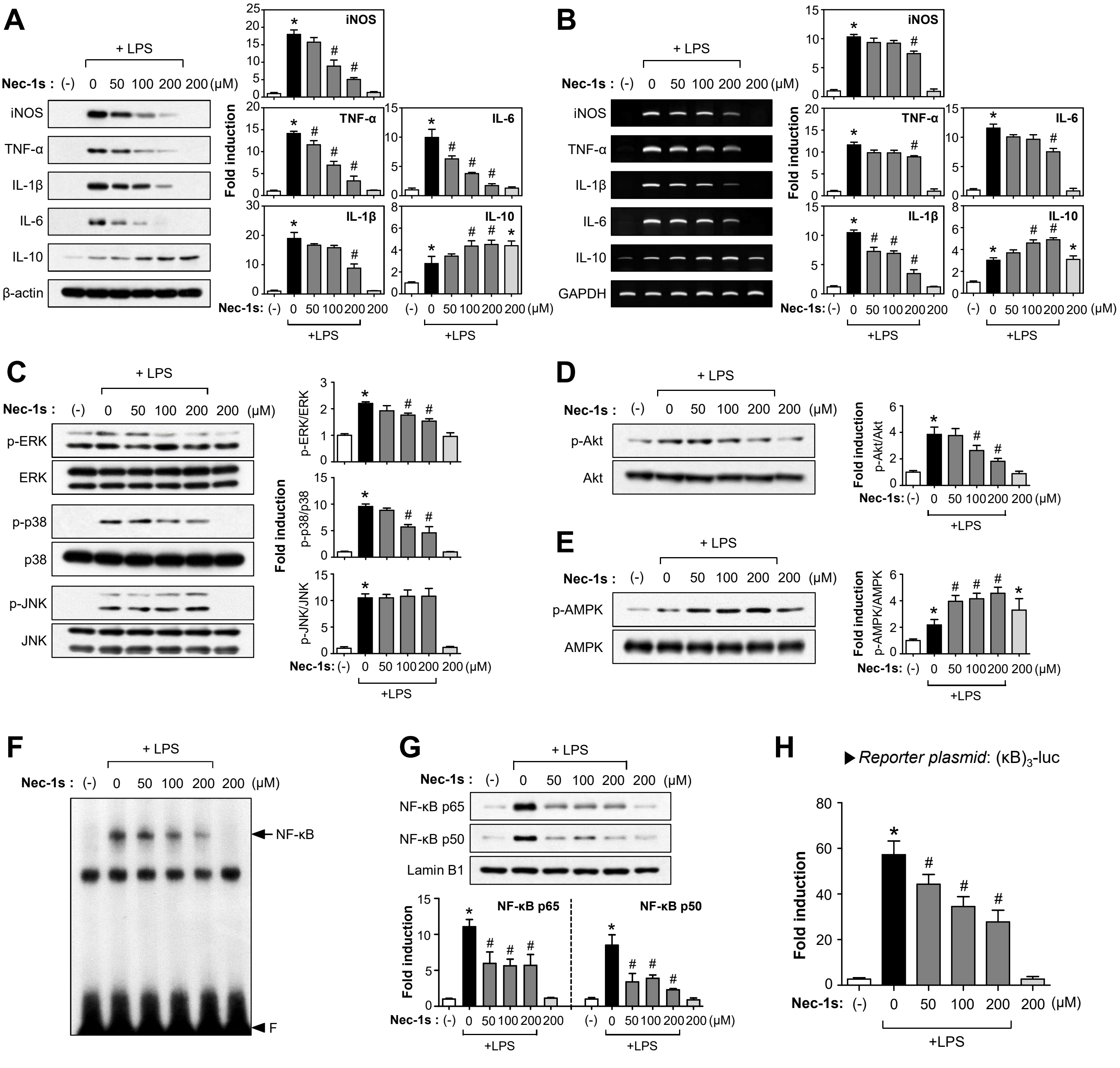

Figure S1. Effect of Nec-1s on inflammatory cytokines and signaling molecules such as MAPKs, Akt, AMPK, and NF-κB in LPS-stimulated BV2 cells. (A, B) BV2 cells were pretreated with Nec-1s for 1 h and incubated with LPS (100 ng/ml) for 6 h. Western blot analysis (A) and RT-PCR (B) were performed to measure the expression of TNF-α, IL-1β, IL-6, IL-10. **(C)~(E)** BV2 cells were pretreated with Nec-1s and incubated with LPS (100 ng/ml) for 1 h. Western blot analysis using antibodies against the phospho- or total forms of ERK, p38, JNK, Akt, AMPK were normalized with respect to the level of each total form and expressed as fold changes relative to the control group. (A)~(E), representative gels are shown in the left panel, and the quantification of three independent experiments is shown in the right panel. **(F)** EMSA for NF-κB was performed using nuclear extracts prepared from BV2 cells pretreated with Nec-1s and incubated with LPS (100 ng/ml) for 1 h. **(G)** Effect of Nec-1s on nuclear translocation of NF-κB subunits. **(H)** Transient transfection analysis of $(\kappa B)_3$-luc reporter gene activity. Data are shown as the mean ± SEM of three independent experiments. $^{*}p < 0.05$ vs. control; $^{\#}p < 0.05$ vs. LPS-treated samples.
