# Supplementary material for: RIPK1 Regulates Microglial Activation in Lipopolysaccharide-Induced Neuroinflammation and MPTP-Induced Parkinson’s Disease Mouse Models
Source: Cells. 2023 Jan 26;12(3):417. doi: 10.3390/cells12030417 (PMC9913664; doi:10.3390/cells12030417)
Supplement: Supplementary file 1 [file cells-12-00417-s001.zip › Supplementary Figure S2.pdf]

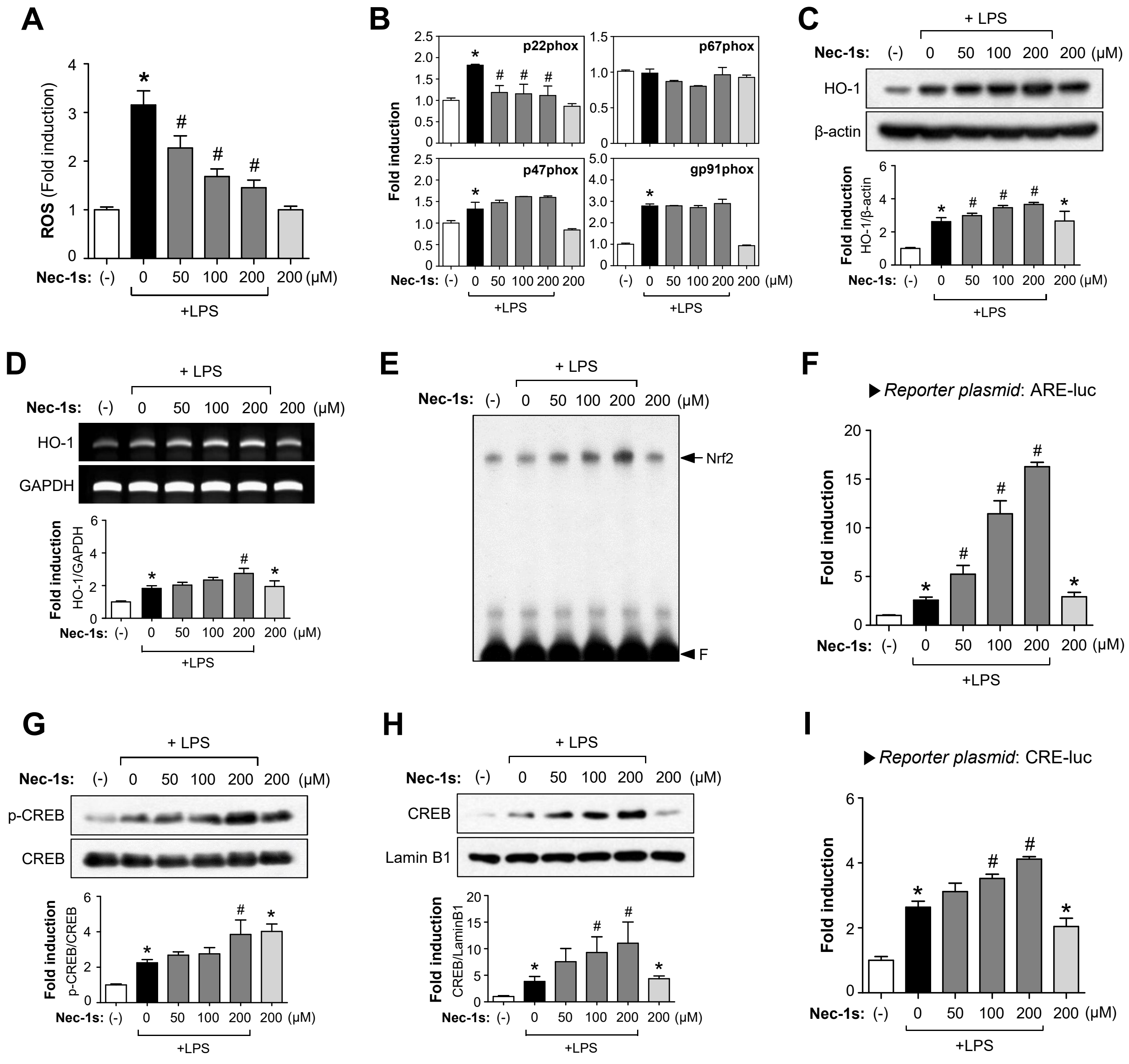

Figure S2. Nec-1s reduced ROS production via suppression of p22phox NADPH oxidase subunit and upregulation of Nrf2/ARE and PKA/CREB signaling. (A) BV2 cells were treated with Nec-1s 1 h prior to LPS stimulation for 8.5 h. Intracellular ROS level was measured using the DCF-DA assay. **(B)** Quantitative real time PCR to assess mRNA expression of NADPH oxidase subunits ($p22^{phox}$, $p47^{phox}$, $p67^{phox}$, and $gp91^{phox}$) in BV2 cells. **(C, D)** Western blot and RT-PCR analysis to determine the effect of Nec-1s on the protein and mRNA expression of HO-1. BV2 cells were treated with Nec-1s for 1 h followed by LPS (100 ng/ml) for 6 h. **(E)** EMSA for Nrf2 DNA binding activity. BV2 cells pretreated with Nec-1s and incubated with LPS (100 ng/ml) for 1 h. **(F)** Transient transfection analysis of ARE-luc reporter gene activity. **(G, H)** Western blot analysis for p-CREB and total CREB was performed using cell lysates and nuclear extracts from BV2 cells, respectively. **(I)** Transient transfection analysis of CRE-luc reporter gene activity. Data are shown as the mean ± SEM of three independent experiments. $^{*}p < 0.05$ vs. control; $^{\#}p < 0.05$ vs. LPS-treated samples.
