# Supplementary material for: RIPK1 Regulates Microglial Activation in Lipopolysaccharide-Induced Neuroinflammation and MPTP-Induced Parkinson’s Disease Mouse Models
Source: Cells. 2023 Jan 26;12(3):417. doi: 10.3390/cells12030417 (PMC9913664; doi:10.3390/cells12030417)
Supplement: Supplementary file 1 [file cells-12-00417-s001.zip › Supplementary Table S1.pdf]

Table S1. List of primary antibodies used in western blot analysis

Antigen	Manufacturer	Catalog number	Host	Dilution
β-actin	Sigma	A1978	Mouse	1:10000
Akt	Cell signaling	9272	Rabbit	1:2000
p-Akt	Cell signaling	9271	Rabbit	1:2000
AMPK	Cell signaling	2603	Rabbit	1:2000
p-AMPK	Cell signaling	2535	Rabbit	1:2000
BDNF	Sigma	SAB2108004	Rabbit	1:2000
CREB	Cell signaling	9197	Rabbit	1:2000
p-CREB	Cell signaling	9198	Rabbit	1:1000
ERK	Cell signaling	9102	Rabbit	1:2000
p-ERK	Cell signaling	9101	Rabbit	1:2000
GDNF	Abcam	ab18956	Rabbit	1:1000
HMGB1	Cell signaling	3935	Rabbit	1:2000
HO-1	Enzo	ADI-SPA-895	Rabbit	1:2000
IL-1β	R&D	AF-401-NA	Goat	1:5000
IL-6	Cell signaling	12912	Rabbit	1:1000
IL-10	Santa Cruz	365858	Mouse	1:1000
iNOS	BD bioscience	610431	Mouse	1:2000
JNK	Cell signaling	9252	Rabbit	1:2000
p-JNK	Cell signaling	9251	Rabbit	1:2000
Lamin B1	Cell signaling	13435	Rabbit	1:1000
MLKL	Cell signaling	37705	Rabbit	1:1000
p-MLKL	Cell signaling	37333	Rabbit	1:1000
NF-kB p65	Santa Cruz	372X	Rabbit	1:5000
NF-kB p50	Santa Cruz	1190X	Goat	1:5000
PGC-1α	Millipore	ST1202	Mouse	1:1000
p38	Cell signaling	9212	Rabbit	1:2000
p-p38	Cell signaling	9211	Rabbit	1:2000
RIPK1	Cell signaling	3493	Rabbit	1:2000
p-RIPK1	Cell signaling	53286	Rabbit	1:1000
RIPK3	Cell signaling	15828	Rabbit	1:2000
p-RIPK3	abcam	209384	Rabbit	1:1000
TH	Cell signaling	58844	Rabbit	1:2000
TNF-α	Cell signaling	11948	Rabbit	1:1000
