# Supplementary material for: RIPK1 Regulates Microglial Activation in Lipopolysaccharide-Induced Neuroinflammation and MPTP-Induced Parkinson’s Disease Mouse Models
Source: Cells. 2023 Jan 26;12(3):417. doi: 10.3390/cells12030417 (PMC9913664; doi:10.3390/cells12030417)
Supplement: Supplementary file 1 [file cells-12-00417-s001.zip › Supplementary Table S2.pdf]

Table S2. List of primary antibodies used in immunohistochemical and immunofluorescence staining

Antigen	Manufacturer	Catalog number	Host	Dilution
CD11b(OX-42)	Bio-rad	MCA711G	Rat	1:500
Iba-1	Wako	019-19741	Rabbit	1:500
p-RIPK1	Cell signaling	53286	Rabbit	1:500
TH	Cell signaling	58844	Rabbit	1:1000
